# DC-shifts in amplitude in-field generated by an oscillatory interference model of grid cell firing

**DOI:** 10.3389/fnsys.2014.00001

**Published:** 2014-01-24

**Authors:** Angela C. E. Onslow, Michael E. Hasselmo, Ehren L. Newman

**Affiliations:** Department of Psychology, Center for Memory and Brain, Boston UniversityBoston, MA, USA

**Keywords:** grid cells, theta phase precession, oscillatory interference model, leaky-integrate-and-fire neuron, oscillations

## Abstract

Oscillatory interference models propose a mechanism by which the spatial firing pattern of grid cells can arise from the interaction of multiple oscillators that shift in relative phase. These models produce aspects of the physiological data such as the phase precession dynamics observed in grid cells. However, existing oscillatory interference models did not predict the in-field DC shifts in the membrane potential of grid cells that have been observed during intracellular recordings in navigating animals. Here, we demonstrate that DC shifts can be generated in an oscillatory interference model when half-wave rectified oscillatory inputs are summed by a leaky integrate-and-fire neuron with a long membrane decay constant (100 ms). The non-linear mean of the half-wave rectified input signal is reproduced in the grid cell's membrane potential trace producing the DC shift within field. For shorter values of the decay constant integration is more effective if the input signal, comprising input from 6 head direction selective populations, is temporally spread during in-field epochs; this requires that the head direction selective populations act as velocity controlled oscillators with baseline oscillations that are phase offset from one another. The resulting simulated membrane potential matches several properties of the empirical intracellular recordings, including: in-field DC-shifts, theta-band oscillations, phase precession of both membrane potential oscillations and grid cell spiking activity relative to network theta and a stronger correlation between DC-shift amplitude and firing-rate than between theta-band oscillation amplitude and firing-rate. This work serves to demonstrate that oscillatory interference models can account for the DC shifts in the membrane potential observed during intracellular recordings of grid cells without the need to appeal to attractor dynamics.

## Introduction

Grid cells recorded in the medial entorhinal cortex (MEC) demonstrate a spatially periodic firing pattern as an animal moves around its environment (Hafting et al., 2005). A grid cell is observed to fire at the vertices of a triangular grid; neighboring grid cells share a similar spatial scale and orientation between their firing patterns but often with some spatial offset (Hafting et al., 2005; Barry et al., 2007). Grids can be seen to rescale or realign in response to visual and spatial changes to a familiar environment (e.g., moving between two rooms, changing a square environment for a circular one or moving the walls of a familiar environment) (Barry et al., 2007; Fyhn et al., 2007), however the intrinsic spatial phase of each grid cell relative to other grid cells is maintained. The body of data on grid cells has lead researchers to suggest that their function is to generate a spatial map of the current environment using a path integration mechanism (McNaughton et al., 2006; Moser et al., 2008).

There are currently two major categories of computational model proposed to underlie grid cell activity: (i) oscillatory interference models, which utilize the phase difference between two oscillators, one of which changes in frequency in proportion to velocity, to allow tracking of distance moved and readout of this information in the amplitude of the interference pattern formed between the oscillators (Burgess et al., 2007; Hasselmo et al., 2007, 2009; Blair et al., 2008; Burgess, 2008), (ii) continuous attractor network models, which use symmetrical recurrent connections between a network of cells to generate an attractor state corresponding to a “bump” of highest activity that can be moved around the network in proportion to distance moved (Fuhs and Touretzky, 2006; McNaughton et al., 2006; Guanella et al., 2007). Whilst there has been some experimental support for both these models (Giocomo et al., 2011) there is still no clear consensus on which is the most likely mechanism of grid cell generation and more recent models have combined elements of both mechanisms (Hasselmo and Brandon, 2012; Navratilova et al., 2012; Hasselmo, 2013).

Recent intracellular recordings made in head fixed mice navigating a virtual reality environment have demonstrated experimental support for a prediction of continuous attractor network models that has not been shown in oscillatory interference models. These recordings showed a distinct increase in the mean DC (direct current) level of the membrane potential voltage fluctuations of a grid cell as the animal moved through the grid cell firing field (Domnisoru et al., 2013; Schmidt-Hieber and Häusser, 2013). These increases in the DC voltage level in-field have been referred to as “ramps” and were shown to be more strongly correlated with location within the grid field than the amplitude of concurrent theta oscillations, though the amplitude of oscillations also showed a weak positive correlation with grid cell firing fields, consistent with the prediction of oscillatory interference models.

**Figure 1:**
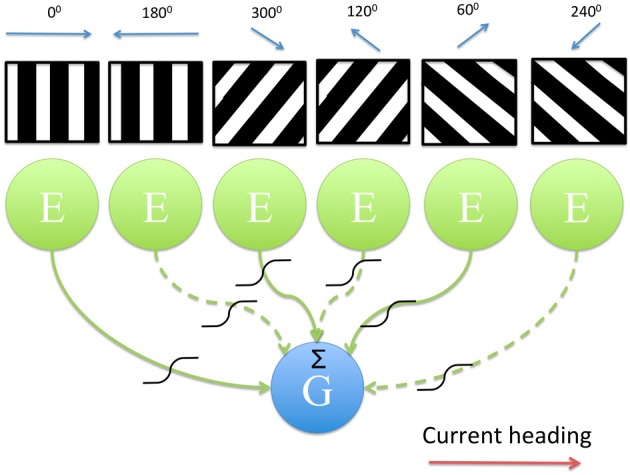
**Diagram showing the model architecture**. 6 directionally tuned band cells, modeled as populations of excitatory neurons, provide input to a leaky integrate-and-fire neuron which models a single grid cell. The band pattern is representative of the firing of each population across the environment; due to the oscillatory interference mechanism employed by each population cells fire periodically as the animal moves in a straight line along a particular heading direction (black, high firing rate activity; white, low firing rate activity). Output from each head direction selective population is subject to a sigmoidal activation function as well as a Heaviside threshold which sets the output of that population to zero if the current heading direction and the preferred heading direction differ by more than 90°. Given the current heading (red arrow) the inputs that differ in their preferred direction from this heading by more than 90° are indicated as dashed lines.

In this paper we present a model that utilizes oscillatory interference as a mechanism for path integration (Burgess et al., 2007; Burgess, 2008) and also produces a shift in the DC voltage level within a grid cell as the animal moves through the grid cell's firing field. The model consists of a leaky integrate-and-fire neuron that receives oscillatory input from head direction selective band cells (HD band cells); these cells act as velocity controlled oscillators (VCOs) (Burgess, 2008) and we refer to them here as band cells because interference between their velocity controlled oscillation and their baseline frequency oscillation produces spatially periodic firing across the extent of the environment, resembling bands that are perpendicular to the head direction selectivity of the cell. Experimental data supports the existence of band cells (Krupic et al., 2012). A schematic of the model is shown in Figure 1. Since the leaky integrate-and-fire neuron sums the oscillatory inputs from 6 HD band cells, with preferred heading directions separated by 60°, its resulting firing pattern is equivalent to the spatial interference pattern formed by overlaying these bands, i.e., peak firing will occur at the vertices of a triangular grid overlaid on the environment.

Oscillatory inputs to the grid cell are half-wave rectified before being summed and this provides the combined input signal with a non-linear DC value, which is reproduced in the grid cell's membrane potential once the input is integrated. In order to best match the empirical data we have used a long decay time constant (=0.1 s). For much shorter decay time constant values (~0.01 s) integration of the HD band cell input signal still results in small sustained DC depolarizations within field, the amplitude of which can be increased if the 6 HD band cell populations deliver their maximal inputs slightly phase offset from one another, so that they are temporally spread during in-field epochs and therefore deliver more consistent large amplitude input to the grid cell during this time. In order to allow for variation in the decay time constant value, the HD band cell populations in our model perform oscillatory interference relative to baseline oscillations that are phase offset from one another, with maximally spread offset values. These three features: a long decay time constant, baseline temporal phase offsets between the HD band cells that act as VCOs and half-wave rectification of HD band cell inputs to the grid cell are the critical additions to previous oscillatory interference models (Burgess, 2008; Schmidt-Hieber and Häusser, 2013) that enable the replication of the observed DC shifts within field. In previous models the oscillatory input to the grid cell had a linear mean or DC value, which produced a similar linear DC value in the grid cell output signal. Without longer integration periods and/or temporal phase offsets between incoming inputs oscillating at theta frequency the troughs (which equate to zero input in the half-wave rectified signals) would lead to a decrease in the membrane potential due to passive leak conductances and make sustained integration and depolarization within field impossible.

The oscillatory output of the grid cell demonstrates phase precession with respect to a baseline theta oscillation, i.e., the cell fires at increasingly early phases of the theta oscillation as the animal moves through a grid field. Phase precession is an important observed feature of grid cells recorded *in vivo* (Hafting et al., 2008) and is not integral to continuous attractor network models, which do not require oscillatory dynamics of individual grid cells [although phase precession can be included in attractor models (Navratilova et al., 2012)].

Our model is explained in more detail in the Materials and Methods section. In the Results section we show that this model produces voltage traces which resemble those recorded by Domnisoru et al. (2013) and Schmidt-Hieber and Häusser (2013) and are in-line with their results despite using oscillatory interference as the path integration mechanism. Finally, we discuss where this model fits in the current understanding of grid cell mechanisms and function.

## Materials and methods

### Modeling

A single entorhinal grid cell is modeled as a leaky integrate-and-fire neuron. This was simulated using Euler integration in MATLAB (2011). The equation used to model the grid cell is given in Equation (1.1). Table 1 gives the default values of all parameters that appear in equations in this section.

(1.1) $$V_{m}(t+1)=V_{m}(t)+\left[I(t)-G_{L}\left(V_{m}(t)-E_{L}\right)\right]dt$$

**Table 1:** **Parameter values of the model, their descriptions and default values**.

**Parameter**	**Description**	**Default value**
τ_GL_	Membrane leak time constant	0.1 s
E_*L*_	Resting potential	−67 mV
*V*_*t*_	Spiking threshold	−56 mV
φ_offset_n_	Baseline phase offset for *n*th HD band cell population (VCO)	[0°, 60°, 120°, 180°, 240°, 300°]
Φ_preferred_n_	Preferred heading direction for *n*th HD band cell population (VCO)	[0°, 60°, 120°, 180°, 240°, 300°]
ω_*b*_	Baseline angular frequency	2π*6 Hz
β	Grid spacing scale factor	0.002 (0.00385 in Figures 4C,D)
a	Sigmoid slope	4
T	Sigmoid inflection point	1
*G*_*I*_	Scaling constant	100

Here *V*_*m*_ is the membrane potential, *G*_*L*_ is the membrane leak conductance (*G*_*L*_ = 1/τ_GL_, where τ_GL_ is the integration time constant = 0.1 s) and E_*L*_ is the resting potential for the cell (*E*_*L*_ = −67 mV). The grid cell fires if *V*_*m*_(*t*) > *V*_*t*_, where *V*_*t*_ = −56 mV. We have not included resetting or refractory dynamics here since these typically occur over a time period of 0.001 s and the time step (*dt*) for our simulations is 0.002 s so these faster dynamics are effectively filtered out. The *I* term consists of summed and scaled oscillatory inputs from 6 populations of head direction selective cells (HD band cells). The output of each of these populations is generated by an oscillatory interference mechanism occurring between oscillations produced by the cells in the population and a baseline oscillation received by each population (the baseline is assumed to be theta frequency input to entorhinal cortex from medial septum). Hence, the HD band cell populations behave as velocity-controlled oscillators (VCOs) (Burgess, 2008). The baseline oscillation that is received by the populations of HD band cells has the same frequency between populations but some temporal phase offset exists between populations (φ_offset_n_). The presence of these baseline phase offsets, combined with the long integration time constant, represent the first two (of three) important features of our model that lead to DC shifts in the grid cell firing fields. The equation governing the dynamics of a single population of HD band cells is given in Equation (1.2). A schematic of the model architecture is shown in Figure 1.

(1.2) $$HD_{n}(t)=S\left\{\cos\left(\omega_{b}t+\varphi_{\text{offset}\_\text{n}}\right)+\cos\left[\omega_{HDn}(t)\right]\right\}\;\cdot \;{\left[\phi_{\text{current}}-\phi_{\text{preferred}\_\text{n}}\right]}_{H}$$

(1.3) $$\omega_{HDn}(t)=\left(\omega_{b}t+\phi_{\text{offset}\_\text{n}}\right)+\beta \;\cdot \;s(t)\;\cdot \text{ cos}\left(\phi_{\text{current}}-\phi_{\text{preferred}\_\text{n}}\right)$$

(1.4) $$S(x)=\frac{1}{1+e^{-a(x-\text{T})}}-\frac{1}{1+e^{a\text{T}}}$$

(1.5) $$H(x)=\left\{\begin{array}{ll} 0, & x>90^{{}^{\circ}}\\ 1, & x\leq 90^{{}^{\circ}} \end{array}\right\}$$

(1.6) $$I(t)=\sum_{n=1:6}G_{I}\;\cdot \;HD_{n}(t)$$

ω_*b*_ is the angular frequency of the baseline oscillation, which in our simulations is set to 2π∗6 Hz (theta frequency) for all results presented here. The model has been tested for several frequencies within the established theta frequency range (6–12 Hz) and the qualitative behavior has been found to be similar (results not shown). φ_offset_n_ is the phase difference in radians that distinguishes the baseline oscillatory input to the *n*th population of HD band cells; these baseline temporal phase offset values were chosen to be equally spaced between 0° and 360°. ω_*HDn*_ is the angular frequency of the oscillation produced by the *n*th HD band cell population and is determined by Equation (1.3). The frequency of this oscillation differs from the baseline oscillation frequency in proportion to the difference between the animal's current heading direction (Φ_current_) and the preferred heading direction of the *n*th population of HD band cells (Φ_preferred_n_). The values of Φ_preferred_n_ were chosen to be spaced at intervals of 60° in order to replicate the triangular grid spacing recorded *in vivo*, therefore our 6 HD band cell populations are selective for the following preferred heading directions [0° (i.e., positive direction on the x-axis), 60°, 120°, 180°, 240°, 300°]. β is a positive constant which can be used to tune the spacing of the grid fields which will be mapped out by the model (β is set to either 0.002 or 0.00385 in our simulations), *s*(*t*) is the animal's running speed. HD band cell activity is scaled by the constant *G*_*I*_, in order that it be of the same magnitude as currents generated by synaptic conductances, and then summed [Equation (1.6)], before it is received as an input current by the grid cell. It should be noted that the output activity of HD band cells is fully sinusoidal rather than periodic spikes, as has been used in other variants of the oscillatory interference model (Hasselmo, 2008).

Equations (1.2) and (1.3) are similar to the oscillatory interference mechanism first proposed by Burgess et al. (2007) [compare Equation (1.3) here to Equation (4) in (Burgess et al., 2007); Equation (1.2) incorporates the interference between a baseline oscillation and a velocity controlled oscillation, see Equation (5) in Burgess et al. (2007)]. The HD band cell populations perform essentially the same computation as the VCOs in Burgess (2008) and interference between the band patterns they create across the environment generates the characteristic grid pattern. However there are some modifications from the VCOs presented in Burgess (2008); firstly, we implement a sigmoid function [*S*(*x*) in Equation (1.4)] to half-wave rectify the oscillations produced by the HD band cell populations. This rectification is the third important addition to this oscillatory interference model, which leads to DC shifts within field. The sigmoid parameter values used are *a* = 4, *T* = 1. The second term on the right hand side of Equation (1.4) ensures that the sigmoid function produces zero output activity when given zero input. Physiologically this rectification can be justified in one of two ways: (i) as replicating the purely positive input that would be received by the grid cell if the HD band cells are excitatory cells, or (ii) as modeling the average firing rate activity of HD band cell populations, each of which only fires when their membrane potential is depolarized above threshold as a result of the oscillatory interference mechanism (i.e., only during peaks of the interference signal and not during troughs).

As an additional modification to the VCOs presented in (Burgess, 2008), the HD band cells are also subject to a threshold based on how similar the current heading direction (Φ_current_) is to their preferred heading direction (Φ_preferred_n_). If the difference between these angles is greater than 90° then the population does not produce discernible firing rate activity since *HD*_*n*_(*t*) is set to zero via the Heaviside function term in Equation (1.2) being equal to zero [refer to Equation (1.5)]. This is similar to the directional VCO model proposed in (Burgess, 2008) and ensures that HD band cells only precess (i.e., increase their frequency) in relation to their baseline oscillation and negates the possibility of procession (a decrease in frequency relative to the baseline oscillation). Since the input to the grid cell [Equation (1.6)] will be the sum of only oscillations with an increased frequency in comparison to the baseline frequency, the membrane potential of the grid cell [Equation (1.1)] will also vary as an oscillation with an increased frequency relative to the baseline oscillation and so demonstrate phase precession. Theta phase precession is an established feature of electrophysiological recordings of entorhinal grid cells (Hafting et al., 2008); grid cells are observed to fire at increasingly earlier phases of the recorded local field potential theta rhythm (Domnisoru et al., 2013) and the peaks of grid cells' membrane potential oscillations recorded intracellularly demonstrate a similar effect relative to local field theta (Schmidt-Hieber and Häusser, 2013).

### Simulated rat trajectory generation

In the simulations shown in Figures 4A,B the simulated rat follows the trajectory of a real rat recorded in our lab (Climer et al., 2013). The data had a time step (*dt*) = 0.02 s which was then up-sampled to 0.002. Position values given as (*x*, *y*) coordinates were used to generate the movement vector at each time point: (Δ*x* = *x*(*t*) − *x*(*t* − 1), Δ*y* = *y*(*t*) − *y*(*t* − 1). A velocity vector was also created as (Δ*x*, Δ*y*)/*dt* and these two vectors provided the input for our simulations.

**Figure 2:**
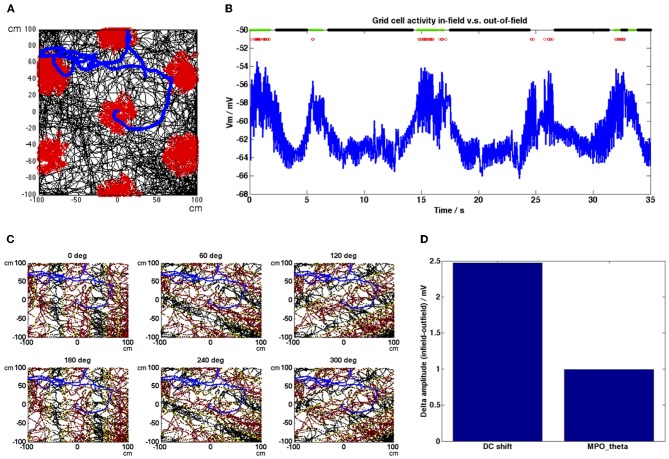
**Grid cell spiking and membrane potential oscillation activity and HD population inputs during a simulated random trajectory. (A)** The trajectory shown in black shows the movement of a simulated rat around a 200 × 200 cm environment. Grid cell spiking activity is shown as red dots along this trajectory, forming a spatially periodic triangular grid. Speed along the trajectory was on average 22.74 cm s^−1^. **(B)** Membrane potential activity of the grid cell during a short section of the simulation shown in **(A)**. The grid cell produces theta (~6 Hz) oscillations with variable amplitude but which in general show an increase in the DC (mean) level within each firing field. Spikes are shown as red dots, in-field classified positions are indicated with green dots, out-of-field classified positions are indicated with black dots (all other positions are unassigned). **(C)** HD band population firing rate activity before it is thresholded based on current heading direction demonstrated periodic bands formed across the environment, perpendicular to each population's preferred heading direction (red, high firing rate activity; yellow, low firing rate activity), section of trajectory shown in 2B is indicated in blue. **(D)** Difference in average DC shift value during in-field positions compared to out-of-field positions (Δamp_*DC*_) = 2.49 mV; difference in average MPO_θ_ oscillation envelope during in-field positions compared to out-of-field positions (Δamp_MPO_) = 0.98 mV.

**Figure 3:**
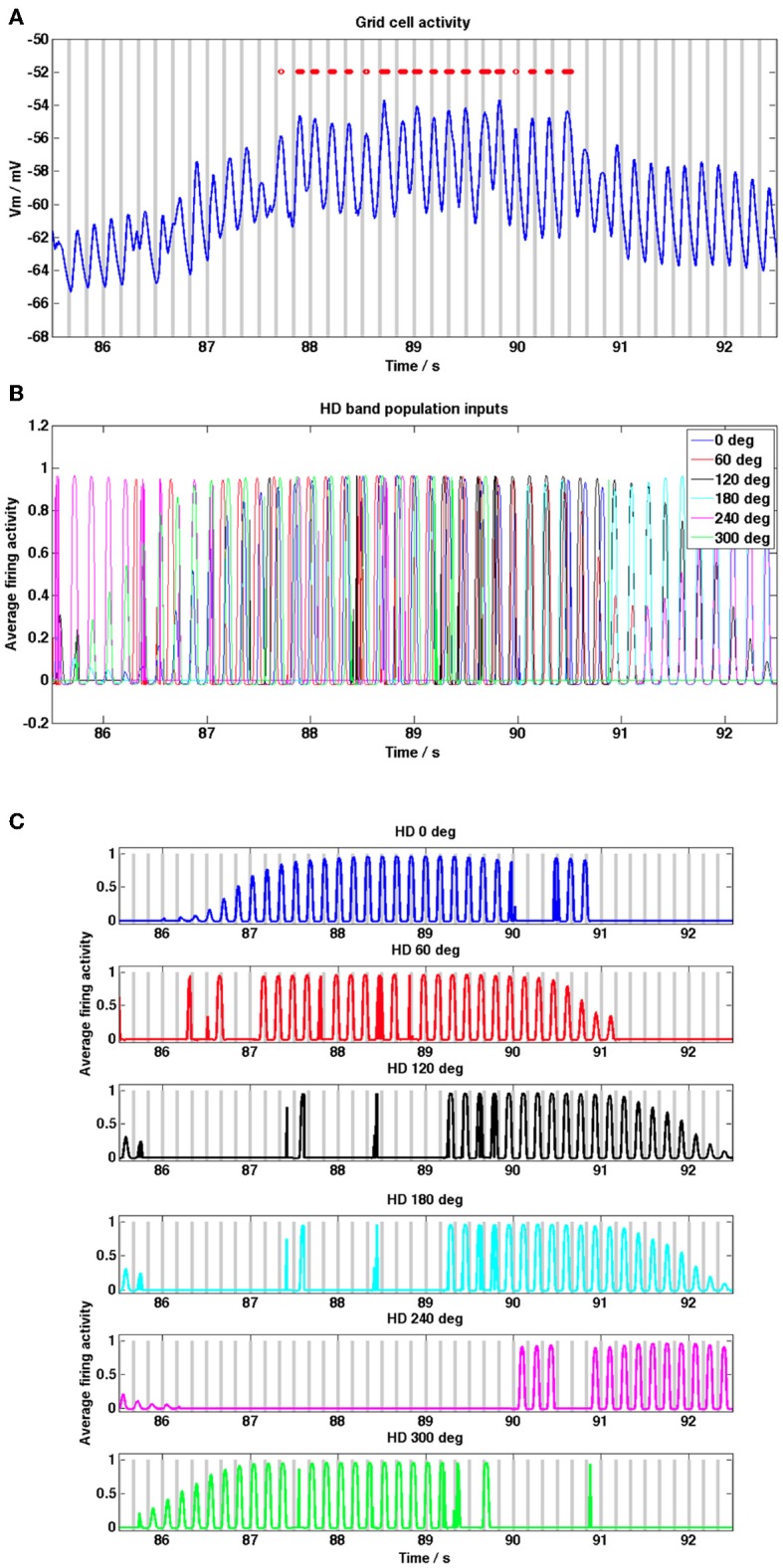
**Grid cell and HD band cell population activity during a grid field crossing, from the random trajectory simulation shown in Figure 2. (A)** Grid cell membrane potential activity (blue trace) within a grid field, baseline theta peaks are shown as gray bars, grid cell spikes are shown as red dots. Phase precession of spikes relative to baseline theta is evident (peaks in membrane potential move from right to left of theta peaks, theta baseline received by the 0° HD population is used as the baseline theta oscillation of the grid cell). **(B)** HD band population firing rate activity, shown for each of the 6 HD band cell populations. **(C)** HD band population activity for each of 6 HD populations, gray bars show theta peaks of the different temporally offset baseline theta oscillation received by each HD band population. During periods of high firing rate activity in each population, i.e., within a band field, theta phase precession of the firing rate activity relative to the baseline theta oscillation can be discerned; since populations increase their firing rate frequency within band fields peaks in the oscillation move from right to left relative to the population's baseline oscillation.

In the simulations shown in Figures 2, 3 a random trajectory was generated using the algorithm provided in (Hasselmo et al., 2007). The components of the movement vector are created as:

(1.7) Δx(t)=s(1−m)p+mΔx(t−1)

(1.8) Δy(t)=s(1−m)p+mΔy(t−1)

Here *s* is the average step size (set to 1.7), *m* is the momentum term [default value is 0.999, initial value is (0.35, 0.35)] and *p* is a random number selected separately for Equation (1.7) and (1.8) at each time step from a normal distribution with mean = 0 and standard deviation = 1. If the simulated rat encounters one of the walls of the enclosure its movement for that time step in the appropriate dimension (*x* or *y*) is generated as:

(1.9) $$\Delta x_{r}(t)=-R\Delta x(t)$$

(1.10) $$\Delta y_{r}(t)=-R\Delta y(t)$$

where *R* is the reverse step size (default = 0.6). This means that the simulated rat moves away from, rather than hugging, the walls of the enclosure.

**Figure 4:**
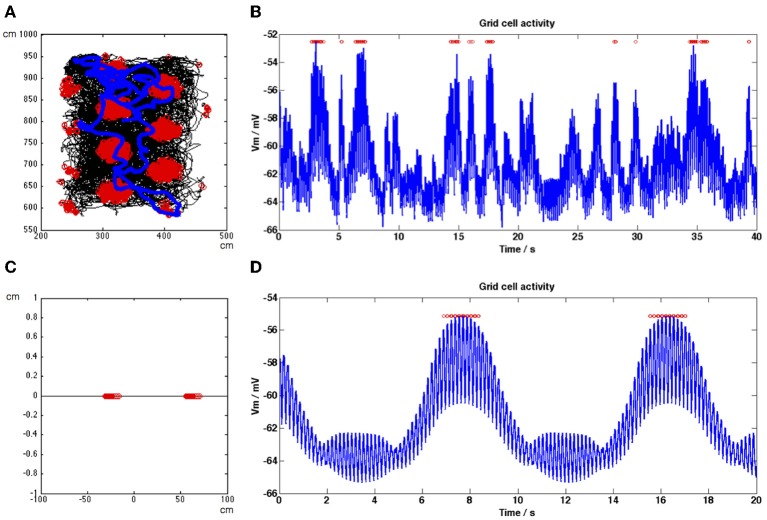
**Firing rate maps and grid cell membrane potential traces generated by the model for a recorded animal trajectory (A,B) and during a straight-line trajectory (C,D). (A)** Trajectory in black shows movement of the rat around a square environment. Grid cell spiking activity generated by the model using this trajectory is shown as red dots. **(B)** Membrane potential activity of the grid cell (blue trace) during a section of the simulation shown in **(A)**, spikes are shown as red dots. The DC level of the trace increases within field, concurrent with spiking activity. **(C)** Straight-line trajectory of a simulated rat is shown in black, grid cell spikes are shown as red dots. For this simulation only β in Equation (1.3) is equal to 0.00385 in order to adjust grid field spacing to view two clear grid fields. **(D)** Membrane potential activity of the grid cell (blue trace) during the entire simulation shown in **(C)**, spikes are shown as red dots. DC level and MPO_θ_ oscillation amplitude increase within grid fields.

**Figure 5:**
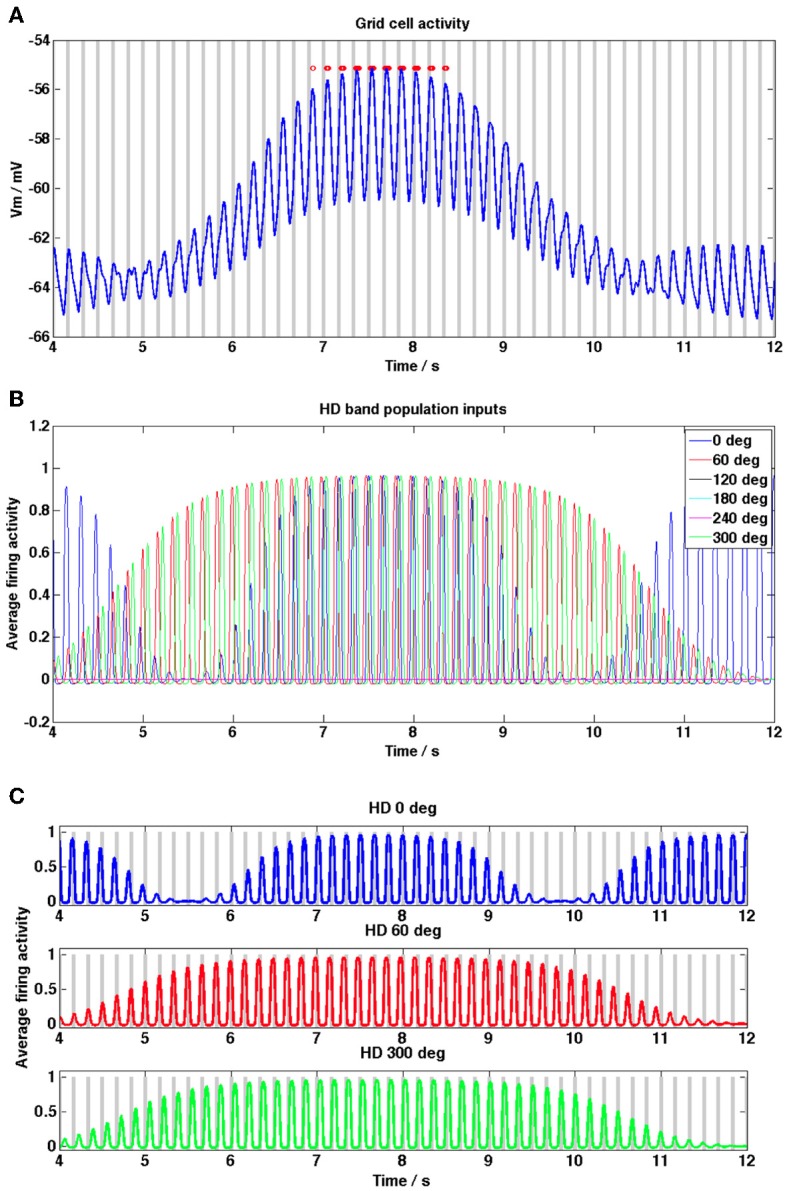
**Grid cell and HD band cell population activity within a grid field during the straight-line trajectory simulation shown in Figure 4D. (A)** Grid cell membrane potential activity (blue trace) within a grid field, baseline theta peaks are shown as gray bars, grid cell spikes are shown as red dots. Notice that the red dots show phase precession relative to the gray bars (i.e., move from right to left, toward earlier theta peaks). **(B)** HD band population firing rate activity, shown for each of the 6 HD band cell populations (although three are set to zero since their preferred heading directions are not within 90° of the current heading direction). **(C)** HD band population activity for each of the three non-zero preferred heading directions; gray bars show theta peaks of the baseline theta oscillation received by each HD band population. Notice that each HD band population's firing rate activity precesses relative to the baseline theta oscillation which that HD band population receives (peaks move from right to left between gray bars).

The simulations shown in Figures 4C,D and 5 were generated using a simulated straight-line trajectory, with (Δ*x*, Δ*y*) beginning at the initial point (−100, 0) in the enclosure space and increasing the x component by 10 (cms^−1^)×*dt* at each time step until the point (100, 0) was reached. For this simulation only β in Equation (1.3) is equal to 0.00385 in order to adjust grid field spacing to view two clear grid fields.

### Data analysis

In order to detect in-field and out-of-field periods we replicated the analysis used for this process in Domnisoru et al. (2013), with some modifications given that our data is 2D rather than 1D. The environment was divided into 5 cm square spatial bins and in-field positions were defined as bins in which the grid cell fired at a higher rate than would be expected by chance, whilst out-of-field positions were defined as bins in which the grid cell fired at a rate lower than would be expected by chance. For each bin a bootstrap shuffled distribution of the grid cell's firing rate was generated by circularly shuffling the entire spike series and then calculating the firing rate using the new spike positions but original trajectory data and therefore time spent in each bin. This process was repeated 1000 times, each time rotating the spike indices by an integer number drawn from a uniform distribution on the range [0.05×total number of data points, 0.95×total number of data points]. A *P*-value was defined for each bin, equal to the number of shuffled firing rates that were higher than the grid cell's actual firing rate for that bin. Out-of-field positions were defined as 2 × 2 adjacent bins demonstrating a real firing rate lower than the 5th percentile of the shuffled distribution (1-*P*-value ≤ 0.05). In-field positions were defined as 3 × 3 adjacent bins demonstrating a real firing rate higher than the 85th percentile of the shuffled distribution (1-*P*-value ≥ 0.85). A candidate firing field could be extended by 1 adjacent bin in all directions if those bins demonstrated real firing rates greater than the 70th percentile of the shuffled distribution (1-*P*-value ≥ 0.70). Bins that did not meet any of these criteria were left unassigned as neither in-field nor out-of-field.

Figure 2D shows the change in amplitude of DC shifts and MPO_θ_ oscillations in-field vs. out-of-field. This was again calculated following the procedure in Domnisoru et al. (2013). First, the DC shift time series was generated by band-pass filtering the grid cell membrane potential trace between 0.1 and 3 Hz using a 2nd order Butterworth filter (this filter was applied in both the forwards and the backwards direction, using the MATLAB function filtfilt, in order to eliminate phase shift effects). The MPO_θ_ oscillation envelope time series was generated by filtering the grid cell membrane potential trace between 5 and 10 Hz, again using a 2nd order Butterworth filter applied in both directions. The envelope of this signal was then calculated using the absolute value of the Hilbert transform. In order to calculate the change in DC shift amplitude in-field vs. out-of-field (Δamp_*DC*_) the average value of the DC shift time series was calculated during out-of-field periods and then subtracted from the original series. The average DC shift amplitude in-field was then calculated as the mean value of this modified DC shift time series during points classified as in-field (DC_in−field_). The average DC shift amplitude out-of-field was calculated as the mean value of out-of-field points (DC_out−of−field_) and was equal to zero due to having been subtracted initially. Δamp_DC_ = DC_in−field_ − DC_out−of−field_. Similarly, the average value of the MPO_θ_ oscillation envelope time series for all in-field points was calculated (MPO_in−field_), as was the average value for all out-of-field points (MPO_out−of−field_) and the change in MPO_θ_ oscillation envelope amplitude in-field vs. out-of-field (Δamp_MPO_) was calculated as Δamp_MPO_ = MPO_in−field_ − MPO_out−of−field_.

**Figure 6:**
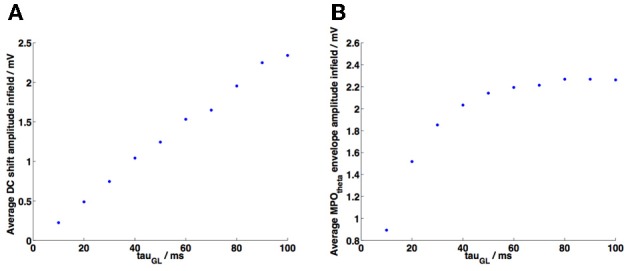
**Parameter analysis of decay constant τ_GL_**. In both plots each point represents the average of 50 random trajectory simulations using different values of τ_GL_ (values of *V*_*t*_ were adjusted accordingly to preserve grid field definition). **(A)** A linear increase was observed in the average value of the maxima of the membrane potential DC shift variation as τ_GL_ was increased. **(B)** The average value of the MPO_θ_ oscillation envelope increased as τ_GL_ was increased, however this began to reach a plateau as τ_GL_ approached the period of one 6 Hz theta cycle (=0.1667 s).

**Figure 7:**
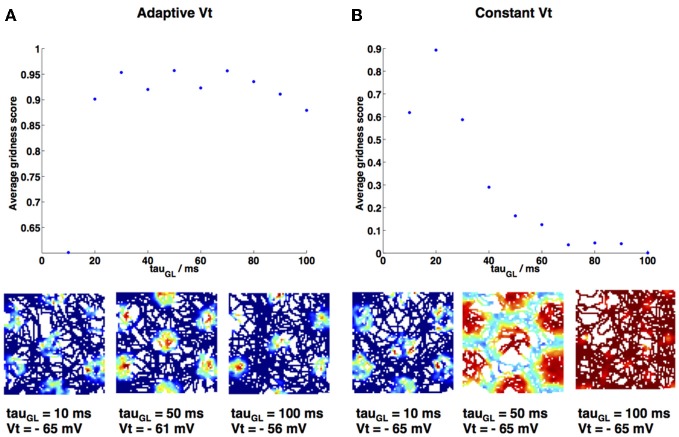
**Gridness score variation as decay constant τ_GL_ is varied. (A)** τ_GL_ is varied and firing threshold Vt is adapted accordingly (values given in Materials and Methods), maintaining a high gridness score. Three example firing rate maps are shown below. **(B)** τ_GL_ is varied and firing threshold Vt is kept constant at −65 mV; grid fields swell and eventually firing is constant over the entire environment (see example firing rate maps below main plot), therefore gridness is lost. Gridness score was calculated using gridness measure 2 from Brandon et al. (2011).

The analysis of the parameter τ_GL_ shown in Figure 6 was conducted as follows: each data point is the average value of 50 data points generated using randomly generated trajectories, each 10 min long. After each simulation DC_in−field_ and MPO_in−field_ were calculated as described above, averaged over the 50 simulations and plotted. As τ_GL_ was varied it was necessary to proportionally increase the value of the spiking threshold *V*_*t*_, in order to maintain grid fields and prevent the model from spiking at every point in the simulation (refer to Figure 7). The following values of τ_GL_ and corresponding values of *V*_*t*_ were used: τ_GL_ = [10,20,30,40,50,60,70,80,90,100] ms, *V*_*t*_ = [65,64,63,62,61,60,59,58,57,56] mV.

**Figure 8:**
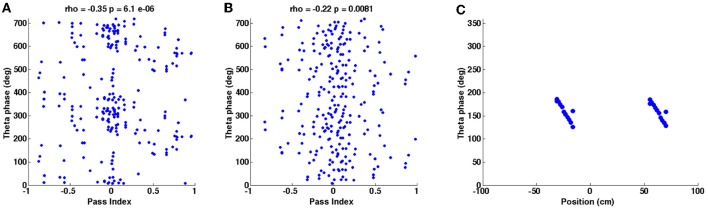
**Phase precession plots for the simulations shown in Figures 2, 4. (A)** Instantaneous phase of baseline theta oscillations is plotted against the animal's position along a field pass (“pass index”) for each in-field spike during the random trajectory simulation shown in Figure 2B. “Rho” is the circular-linear correlation coefficient, given with its associated *p*-value. **(B)** Plot generated as for **(A)** but using data from the real trajectory simulation shown in Figure 4B. **(C)** Instantaneous phase of baseline theta oscillation during spike events is plotted against position of spike events during the straight-line trajectory simulation shown in Figure 4D.

The phase precession plots shown in Figures 8A,B in the Results section were generated by first calculating how “in-field” an animal is at any point along its trajectory using the grid cell firing rate map. Specifically the “field index” was calculated following (Climer et al., 2013), by first creating an occupancy-normalized firing rate map for 1 × 1 cm bins across the environment space. This data was then smoothed using a two-dimensional convolution with a pseudo-Gaussian kernel, with a 5-pixel (equivalent to 5 cm) standard deviation. The firing rate value in each bin was then normalized to between 0 and 1 to produce the “field index map.” For each data point along an experimental trajectory the nearest positional bin was found by minimizing the distance between the (*x*, *y*) position of that point and the center of each bin, using the MATLAB function bsxfun. The field index at each point along a trajectory is then the value of the field index map for the appropriate positional bin.

The angle of the Hilbert transform was then computed on this “in-field” time series in order to extract phasic information regarding how much of a full pass cycle has been completed at any given point; this value is referred to as the pass index (Climer et al., 2013). The corresponding instantaneous phase value of the theta frequency baseline oscillation was then computed at every point (using the angle of the Hilbert transform of the baseline oscillation signal) and plotted against pass index as a scattergram. The baseline theta oscillation received by the 0° selective HD population was used as the baseline oscillation against which phase precession was calculated. A value of the circular-linear correlation, “rho,” is computed for each scattergram following the method detailed in Kempter et al. (2012). Figure 8A was generated using 80s of simulation data (producing 155 spikes) and Figure 8B was generated using 120s of simulation data (producing 143 spikes).

The phase precession plot shown in Figure 8C was generated by calculating the instantaneous phase of baseline theta (using the angle of the Hilbert transform) corresponding to any grid cell spike events. As before, the baseline theta signal received by the 0° selective HD population was used. These theta phase values were then plotted against position along the straight-line trajectory at which each spike event occurred. In all of the phase precession plots in Figure 8 only spike events that occurred closest to the peak of a concurrent grid cell membrane theta cycle were used in the analysis, although the model does permit more than one spike to occur within a grid cell theta cycle so long as the membrane potential is above threshold.

**Figure 9:**
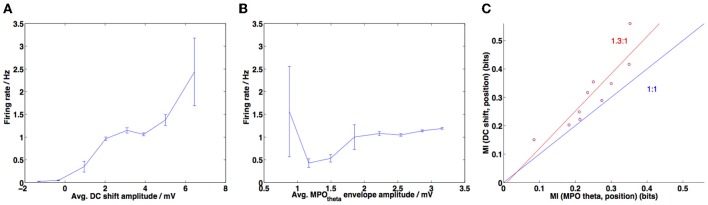
**DC-shift amplitude correlates most strongly with firing rate and provides more information about in-field position. (A)** Amplitude of the DC-shift variation was calculated for each interspike interval during a simulation, along with the corresponding firing rate (=1/interspike interval). This was repeated for 10 simulations of random trajectories (each 1 min in duration) and data points were grouped into 8 bins spanning the range of DC-shift amplitudes. The average firing rate for each bin is plotted here and error bars show the standard error of the mean (s.e.m.) (large error bars correspond to sparsely populated bins). Pearson's correlation coefficient *r* = 0.4278, *p*-value < 10^−10^. **(B)** Amplitude of the MPO_θ_ oscillation was calculated for each interspike interval, for 10 simulations **(**as in **A)** and then the firing rate was averaged across 8 bins spanning the range of MPO_θ_ oscillation amplitudes. Error bars show s.e.m. for each bin. Pearson's correlation coefficient *r* = 0.2644, *p*-value = 7.0605 × 10^−10^. **(C)** MI (mutual information) calculated between DC-shift amplitude or MPO_θ_ oscillation amplitude and the “field index” measure (the latter measures how “in-field” a given position is, refer to Materials and Methods for details). Information contained in DC-shift amplitude is greater than that contained in MPO_θ_ oscillation amplitude (points lie above the blue line indicating a ratio of 1:1, mean ratio of MI values observed is 1.3, shown in red).

The average amplitude of the DC-shift in the grid cell's membrane potential and the average amplitude of the membrane potential theta oscillations were both correlated with the grid cell's firing rate in order to compare their values in-field (corresponding to periods of high firing rate) and out-of-field (corresponding to periods of low firing rate). This analysis was used to produce Figures 9A,B in the Results section and follows the same procedure detailed in Schmidt-Hieber and Häusser (2013) (our Figures 9A,B can be compared to Figure 6A in that paper). First, the firing rate for the grid cell was calculated as the reciprocal of the interspike interval (ISI). As we did for the plots shown in Figure 8, only the spike event closest to each above-threshold peak of the membrane potential theta oscillation was kept in the spike time series and the rest discarded, in order to more faithfully replicate the spike time distributions observed in the empirical data. To generate the DC-shift time series the mean of the membrane potential trace was removed and the resulting signal was band-pass filtered between 0.1 and 3 Hz using a 2nd order Butterworth filter (this filter was applied in both the forwards and the backwards direction, using the MATLAB function filtfilt, in order to eliminate phase shift effects). To generate the theta oscillation amplitude time series the membrane potential trace (minus the mean) was filtered between 5 and 10 Hz and then the envelope was calculated as the absolute value of the Hilbert transform of this filtered signal. The average value of these two time series was found for each ISI observed during 10 simulations using different random trajectories, each 1 min in duration. These results were then binned into 8 bins spanning the ranges of average DC-shift and average theta amplitude values. The average firing rate for each bin was then plotted on a scattergram. Pearson's correlation coefficient was calculated on the unbinned data using the MATLAB function corrcoef.

Figure 9C shows the mutual information (MI) measure calculated between the DC-shift amplitude and the position within field (measured as the “field index”) and between the membrane potential theta oscillation amplitude and position within field. The DC-shift amplitude and MPO_θ_ oscillation amplitude time series were both calculated as described above for Figures 9A,B. The field index time series was calculated following (Climer et al., 2013) and as described above for Figure 8.

**Figure 10:**
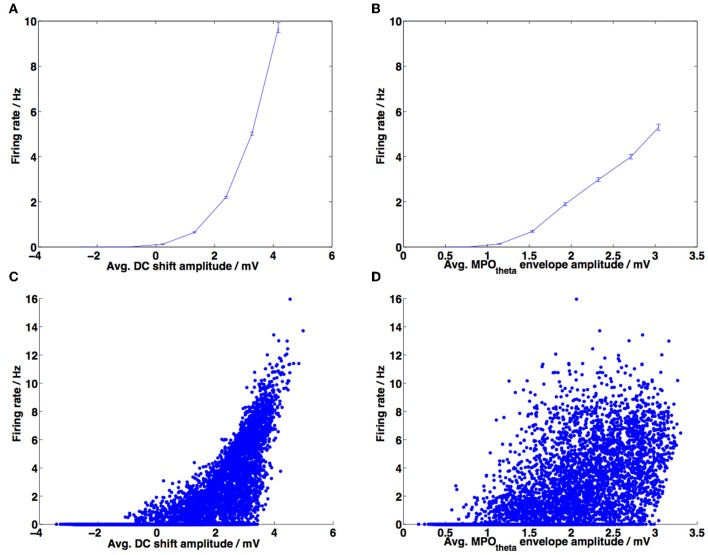
**Correlations between DC-shift amplitude, membrane potential theta (MPO_θ_) amplitude and firing rate. (A)** Amplitude of the DC-shift variation was calculated for 1 s duration sliding windows, along with the corresponding firing rate for each window, normalized by the average firing rate for that experiment. Data from 20 experiments, each 10 min long, are averaged across 8 bins spanning the x-axis. Error bars show the standard error of the mean (s.e.m.). **(B)** Amplitude of MPO_θ_ oscillation was calculated for 1 s duration sliding windows, along with the corresponding firing rate for each window, normalized by the average firing rate for that experiment. Data from 20 experiments, each 10 min long, are averaged across 8 bins spanning the x-axis. Error bars show SEM. **(C)** Unbinned data used to produce plot **(A)** are shown as a scattergram. Pearson's correlation coefficient calculated on this unbinned data: *r* = 0.7068, *p*-value < 10^−10^. **(D)** Unbinned data used to produce plot (B); Pearson's correlation coefficient calculated on this unbinned data: *r* = 0.6375, *p*-value < 10^−10^.

Early investigations showed that the method of examining firing rate as the reciprocal of the ISI meant that the analysis shown in Figures 9A,B was dominated by data points representing in-field periods since this is when the ISI is short and so more data points representing these periods are generated. This problem was reduced when we reduced the number of spike events to only those occurring closest to a theta peak (as described above for Figures 8, 9), however we also implemented a variation on the firing rate analysis shown in Figure 9. Instead of looking at the average DC-shift or MPO_θ_ oscillation envelope during each ISI we looked at these average values within a 1 s duration sliding window. Firing rate was calculated as the number of spikes within that sliding window and average DC-shift or MPO_θ_ oscillation values were then plotted against firing rate and unbinned correlations calculated using the MATLAB function corrcoef. This analysis is shown in Figure 10.

## Results

### DC-shifts within field

Figures 2, 3 show grid cell and HD band population activity during a simulated random trajectory around a 200 × 200 cm square environment. The simulated animal moves at an average speed of 22.74 cm s^−1^ and the total length of the simulation was 2000 s. Figure 2A shows the trajectory in black and the grid cell spiking activity superposed as red dots, forming the typical triangular grid pattern across the environment. The section of the trajectory marked in blue corresponds to the section of grid cell membrane potential activity shown in Figure 2B. Here, again, spikes are represented as red dots and positions which were classified as in-field are marked at the top of the plot with green dots, whilst positions classified as out-of-field are marked with black dots. The amplitude of the theta oscillations produced by the grid cell during in-field positions is somewhat variable, however there is a clear trend for the DC value of the potential to increase within field. Figure 2C shows the firing rate activity of each of the 6 HD band populations before they are thresholded based on the current movement direction, to demonstrate the periodic bands which are formed across the environment, perpendicular to the preferred heading direction. The trajectory is shown in black, red dots correspond to high firing rates and yellow dots correspond to low firing rates (the data was down-sampled to increase clarity; red dots appear flanked by yellow dots on either side as a field band is traversed). The section of the trajectory corresponding to Figure 2B is marked in blue. Figure 2D indicates that the difference in the average DC shift value in-field compared to out-of-field (Δamp_DC_) is larger than the difference in the average MPO_θ_ oscillation envelope value in-field vs. out-of-field (Δamp_MPO_); Δamp_*DC*_ was 2.49 mV whilst Δamp_MPO_ was 0.98 mV. These values compare reasonably well with the experimental results of Domnisoru et al. (2013), who reported a Δamp_*DC*_ value of 2.9 ± 0.3 mV across the cells they observed and a Δamp_MPO_ value of 0.72 ± 0.12 mV.

Figure 3A shows the grid cell membrane potential during a small section of the 2000 s long simulation corresponding to one field crossing. In this diagram peaks in the baseline theta oscillation (taken to be the baseline oscillation received by the HD band cell population receptive for the 0° heading direction but any of the 6 baseline oscillations would suffice) are shown as gray bars and again spikes are shown as red dots. Notice that the grid cell demonstrates phase precession within the field, since peaks in the trace move progressively toward earlier theta peaks (i.e., peaks and spikes move from the right side of each theta peak toward the left as the animal traverses the field). There is a clear increase in the DC membrane potential level when the animal enters a field and spiking activity begins. Figure 3B shows the HD band cell population activity [refer to Equation (1.2)] for the same segment of the simulation as Figure 3A. Figure 3C shows the firing rate activity of each of the 6 HD band cell populations, along with the peaks in the theta baseline oscillation received by each population (gray bars). The oscillatory firing rate activity of each population can be seen to precess (increase in frequency) relative to that population's baseline oscillation within the grid field (oscillation peaks move progressively left relative to baseline peaks). Notice in Figure 3B that within field there is some temporal spread between the contributing oscillatory inputs to the grid cell, due to the temporal phase offsets that exist between each population's baseline theta rhythm [refer to Equation (1.2)]; this provides more consistent input to the grid cell, aiding integration.

To demonstrate that these results are not due to the properties of the simulated behavioral trajectory, Figures 4A,B show the firing rate map and a short section of the grid cell membrane potential trace generated by our model using a real behavioral trajectory recorded from an animal in our lab (Climer et al., 2013). The entire simulation was 2340 s in length. In Figure 4A the trajectory is shown in black with red dots indicating spikes and the short section of the grid cell's membrane potential trace shown in Figure 4B is indicated by the blue trajectory line. The membrane potential trace shown in Figure 4B demonstrates DC-shifts when the simulated animal makes a grid field crossing (spikes shown as red dots). The grid cell membrane theta oscillations tend to be large amplitude within field but they can also decrease during in-field positions (N.B. the 35 s time point), however firing will be maintained if the DC shift amplitude remains high.

In order to provide more intuition for how our model works Figures 4C,D show the firing rate map and a short section of the grid cell membrane potential trace generated by our model using a simulated straight-line trajectory 200 cm in length (from left to right along the x-axis). For this simulation only β in Equation (1.3) was set equal to 0.00385 in order to adjust grid field spacing to view two clear grid fields. In Figure 4C the trajectory is shown in black, in Figure 4D the grid cell membrane potential for the entire trajectory is shown in blue and in both figures spikes are shown as red dots. The simulated animal travelled at a constant speed of 5 cms^−1^, leading to increases in theta amplitude and DC level that are periodic in time as well as space (compare Figures 4C,D).

Figure 5 shows example grid cell and HD band cell population activity during a single pass through a grid field on the straight-line trajectory. In Figure 5A peaks in the baseline theta oscillation are shown as gray bars and spikes are shown as red dots (the baseline theta oscillation received by the 0° heading direction population is shown). Notice that the grid cell demonstrates phase precession within the field, since the spikes move progressively toward earlier theta peaks (i.e., spikes move from the right side of each theta peak to the left as the animal traverses the field). Figure 5B shows the HD band cell population activity [refer to Equation (1.2)] for the same within-field segment of the simulation as 5A. Since the trajectory is straight along the x axis only 3 of the 6 HD band cell populations have preferred heading directions within 90° of this heading and contribute input to the grid cell, the rest have zero level activity. The increase in the DC level of the grid cell corresponds to the time when all 3 HD band populations have high amplitude oscillations; during this time the combination of a long integration time constant of the grid cell membrane (τ_GL_) and the temporal spread between the oscillatory inputs from HD band populations (φ_offset_n_) allows the grid cell to integrate and increase its DC value. With τ_GL_ set to the current long value of 100 ms the temporal phase offsets between the HD band populations can all be set to zero and the grid cell will still produce DC shifts within field (results not shown), however if τ_GL_ is decreased then the role of φ_offset_n_ becomes important in order to observe DC shifts. Large amplitude theta oscillations correspond to times when at least one HD band population produces high amplitude oscillations. When the oscillations produced by different HD band populations are more synchronized, the theta oscillations replicated in the grid cell output become larger.

Figure 5C shows the firing rate activity of each of the 3 HD band cell populations that produce non-zero activity during this simulation, along with the peaks in the theta baseline oscillation received by each population (gray bars). The oscillatory firing rate activity of each population can be seen to precess (increase in frequency) relative to that population's baseline oscillation within the grid field. Comparing Figures 5A–C highlights how large DC-shift values correspond to large amplitude, temporally spread HD population inputs to the grid cell (between 7 and 8 s). Small amplitude grid cell theta oscillations correspond to small amplitude, incoherent (unsynchronized) HD population inputs (between 10 and 11 s). Grid cell theta oscillations can be seen to increase in amplitude when there are several high amplitude theta frequency inputs to the grid cell (between 7 and 8 s) but also when there is only 1 large amplitude theta frequency input (between 11.5 and 12 s), since this corresponds to a large amplitude, coherent input.

### Decay time constant (τ_GL_) parameter analysis

The grid cell membrane decay time constant is a crucial parameter in order to observe DC shifts within field. We conducted a parameter analysis of τ_GL_ in order to examine its effects on the size of DC shifts and of membrane potential theta oscillations within field. The results are shown in Figures 6A,B. As τ_GL_ increases the average value of the maxima of the DC shift variation increased linearly, i.e., larger DC shift values are observed within field (Figure 6A). Initially an increase in τ_GL_ also leads to an increase in the average amplitude of MPO_θ_ oscillations (Figure 6B), however the amplitude of MPO_θ_ oscillations approaches a plateau. This is due to the fact that each theta cycle that appears in the grid cell membrane potential is the result of summing ~3 HD band cell population theta frequency inputs; once τ_GL_ and thus the window of integration has reached the period of a single cycle of these theta frequency inputs (=0.1667 s for the 6 Hz theta oscillations used in our model) then the number of inputs being received by the grid cell within this window becomes the limiting factor. This reaches a maximum when 3 HD band cell populations provide close-to-synchronized inputs to the grid cell.

As τ_GL_ was varied it was necessary to proportionally increase the value of the spiking threshold *V*_*t*_, in order to maintain grid fields and prevent the model from spiking at every point in the simulation. This necessity is demonstrated in Figure 7, which shows the effect on gridness score of increasing τ_GL_ when *V*_*t*_ is either adapted (Figure 7A) or kept constant (Figure 7B). If *V*_*t*_ is kept constant then gridness score decreases as τ_GL_ increases, since the increased integration means that the grid cell begins to spike at every point in the environment. *V*_*t*_ values were chosen to increase in proportion to the increase in τ_GL_ (for every 10 ms increase in τ_GL_ *V*_*t*_ was raised by 1 mV); this produced similar numbers of spikes and gridness scores (Figure 7A) for each simulation as τ_GL_ was varied.

### Theta phase precession

Figure 8 shows the theta phase precession effect that is present during each of the simulations shown in Figures 2, 4. Figure 8A shows the phase precession that appears during the simulation shown in Figure 2 (random trajectory), Figure 8B looks at the phase precession demonstrated by the simulation shown in Figures 4A,B (real trajectory) and Figure 8C looks at phase precession shown by the simulation in Figures 4C,D (straight-line trajectory). In Figures 8A,B the pass index refers to the position of the animal within a grid field relative to a complete pass through the field and this is plotted against the instantaneous phase of the baseline theta oscillation (received by the 0° HD population) for each in-field spike recorded. The circular-linear correlation coefficient “rho” is given above each plot along with the corresponding *P*-value; the negative rho values and the sloping gradients of the plots indicate that spikes occur at progressively earlier baseline theta phases as the animal completes each pass through the field. Figure 8B demonstrates weaker phase precession than Figure 7A but similar magnitude rho values were reported from the *in vivo* data recordings this trajectory was taken from Climer et al. (2013). Figure 8C takes a different approach and plots the instantaneous phase of the 0° HD baseline theta oscillation during each spike event against the position of the simulated animal during that spike event [this replicates the phase precession plots generated for the experimental data in Domnisoru et al. (2013) and Schmidt-Hieber and Häusser (2013)]. Again, it is evident that the model demonstrates phase precession, since spikes occur at progressively earlier theta phases as the animal progresses along the straight-line trajectory.

### Correlations between DC-shift amplitude, theta oscillation amplitude and firing rate

In order to compare our model to the results obtained by Schmidt-Hieber and Häusser (2013) we looked at whether the increase in DC value correlated more closely with high firing rates (i.e., with in-field positions) than the amplitude of grid cell membrane potential theta oscillations. We followed the same procedure for calculating the average DC-shift and average MPO_θ_ oscillation for each ISI as in the previous experimental paper (Schmidt-Hieber and Häusser, 2013) (refer to Materials and Methods). The firing rate was calculated as 1/ISI and the average DC shift and MPO_θ_ oscillation values were collected over 10 simulations, then binned and plotted against the average firing rate for each bin as a scattergram. The results are plotted in Figures 9A,B.

Figure 9A demonstrates that higher DC-shift values are positively correlated with increased firing rates (taken to be equivalent to in-field status of the animal). The Pearson's correlation coefficient (*r*) is 0.4278 (*p*-value < 10^−10^). This compares reasonably well with the results of Schmidt-Hieber and Häusser (2013), although they reported a larger positive correlation coefficient value of 0.99.

Figure 9B demonstrates that the amplitude of grid cell membrane potential theta oscillations also shows a positive correlation with increased firing rate or in-field status (*r* = 0.2644, *p*-value = 7.0506 × 10^−10^). As with the DC-shift vs. firing rate correlation, this value is lower than the positive correlation reported for the experimental data (Schmidt-Hieber and Häusser, 2013) (*r* = 0.57, *p*-value = 0.14) and the positive correlation in the experimental data was not found to be significant. It should be noted that the correlations we have reported are calculated using the unbinned data; this could potentially explain the difference in magnitude and significance between our results and those reported for the experimental data. If we calculate the correlation using the binned data we obtained the following results: for the DC-shift correlation *r* = 0.9567, *p*-value = 0.0002, for the MPO_θ_ oscillation correlation *r* = 0.2242, *p*-value = 0.5935. These binned correlation values are more similar to those reported by Schmidt-Hieber and Häusser (2013), with the DC-shift correlation being larger and statistically significant and the MPO_θ_ oscillation correlation being positive but failing to reach significance. Schmidt-Hieber and Häusser (personal communication) confirmed that when correlations are calculated on their data using unbinned values both correlations are positive and significant but the DC-shift correlation is higher (DC-shift correlation *r* = 0.3639, *p*-value = 2.5392 × 10^−27^, MPO_θ_ oscillation correlation *r* = 0.1220, *p*-value = 0.0004). The difference between the unbinned correlation of DC-shift amplitude with firing rate and the unbinned correlation of MPO_θ_ oscillation envelope with firing rate was found to be statistically significant (Steiger's *Z*_*H*_ = 3.41, *p*-value <0.001).

Figure 9C compares the information regarding in-field position contained within the DC-shift amplitude time series and within the MPO_θ_ oscillation amplitude time series. This is represented as the MI, calculated between each of these time series and the “field index”—a measure of how “in-field” the simulated animal is, which varies between 0 (out-of-field) and 1 (at the center of a field). The information contained within the DC-shift amplitude time series is always larger then that contained within the MPO_θ_ oscillation amplitude time series, as demonstrated by all points lying above the line with ratio 1:1 (blue line). The mean MI ratio of the data is 1.3:1 (red line). This can be compared to Figure 5b in Domnisoru et al. (2013), which also shows an MI ratio greater than 1:1 (=2.5:1).

An issue that arises when analyzing the *in vivo* experimental data is that different neurons can have very different spiking rates and so the ISI analysis suffers from neurons with a higher ISI having a stronger effect on the linear regression. Binning the data can reduce this problem. The MI analysis does not suffer from this issue since each data point, representing the MI for a single neuron (or simulation in our case), is evaluated individually as having more information contained in either the DC-shift time series or in the MPO_θ_ oscillation envelope time series using the same number of spikes. We have also considered a third type of analysis, which, instead of looking at the average DC-shift or MPO_θ_ oscillation envelope during each ISI, looks at these average values within a 1 s duration sliding window. Firing rate is calculated as the number of spikes within that sliding window and average DC-shift or MPO_θ_ oscillation are plotted against firing rate and then the unbinned correlation is calculated. If traces recorded from different neurons (or simulations) are the same length of time then they will contribute the same number of data points to the linear regression. This analysis again shows that both correlations are positive and significant but that the DC-shift correlation value is higher (shown in Figure 10). The difference between the correlation of DC-shift amplitude with firing rate and the correlation of MPO_θ_ oscillation envelope with firing rate was found to be statistically significant (Steiger's *Z*_*H*_ = 13.02, *p*-value <0.001).

## Discussion

We have presented a variant of the oscillatory interference model of grid cell firing that generates DC-shifts. This model was constructed as a leaky integrate-and-fire neuron which sums input from head direction modulated cells that utilize oscillatory interference to track distance moved along a particular heading. Our model addresses the recent discovery from intracellular *in vivo* recordings that grid cell membrane potentials increase their mean DC value within grid fields (Domnisoru et al., 2013; Schmidt-Hieber and Häusser, 2013). This feature was not present in previous models using oscillatory interference as a mechanism for path integration but was a feature of a separate class of path integration models: continuous attractor network models. We are able to demonstrate similar increases in the DC membrane potential value within field in our model (see Figures 2–5), which is similar in structure to the oscillatory interference model proposed by (Burgess, 2008). Our results suggest that the oscillatory interference mechanisms of path integration cannot be ruled out as possibly underlying grid cell formation based on the evidence showing increases in the mean level of grid cells' membrane potentials within field. Some previous variants of the oscillatory interference model did not explicitly simulate membrane potential dynamics [but see one of the model variants presented in Burgess (2008) as well as Zilli and Hasselmo (2010)]. Our model serves to demonstrate that with the inclusion of simple membrane dynamics, incorporating a long decay time constant and combined with baseline temporal phase offsets and half-wave rectification of inputs, in-field DC-shifts can be a feature of the oscillatory interference models of grid cell formation. This is in addition to the previous studies showing that this property would be present in continuous attractor network models of grid cell formation (Domnisoru et al., 2013; Schmidt-Hieber and Häusser, 2013).

DC-shifts in our model arise from the integration of multiple HD inputs to a grid cell with a leak conductance time constant of 100 ms. Since the HD population inputs to the cell are half-wave rectified, the summation of HD inputs by the grid cell can only show increases from the baseline membrane potential value. Combined with the leaky integration mechanism this produces the DC-shifts observed within a grid field (note that at the center of a grid field all contributing HD populations have their highest activity levels so DC increases are largest at these spatial locations). Accordingly, DC-shift amplitude in the model demonstrates a positive correlation with firing rate (as shown in Figure 9A) and demonstrates a difference of approximately 2.49 mV when compared between in-field and out-of-field positions (see Figure 2D), in-line with results observed *in vivo* by Schmidt-Hieber and Häusser (2013) and Domnisoru et al. (2013) respectively. Half-wave rectification of the HD population inputs to the grid cell is an important step in our model, since non-half-wave rectified oscillations would have a constant mean when integrated. Biologically, this rectification would be a natural consequence of the oscillatory interference mechanism being implemented in separate cells or cell populations [as in (Burgess, 2008)] and then these directional velocity-controlled oscillators sending purely excitatory input to the grid cell.

The cells that make up the HD band cell populations are assumed to receive input from various sensory input modalities such as visual, tactile and vestibular inputs, which provide information about the current heading direction and speed. This information is then represented by the selective firing of HD selective cells (Taube et al., 1990a,b; Taube, 2007), which produce an intrinsic theta frequency oscillation when they are activated that is of a slightly faster frequency than the theta frequency input received from medial septum (the latter provides the baseline oscillation in our model). An interference pattern is generated when an individual cell sums these two oscillatory input patterns. Several cells are assumed to be selective for the same heading direction and strong connections between these cells would act to synchronize their activity and allow them to act as a functional ensemble, in-line with the population level modeling approach we have taken. Cells within a particular HD selective population receive the same baseline frequency input and generate synchronized intrinsic oscillations whilst any differences in their threshold firing level are taken into consideration via the sigmoidal activation function applied to the population's output [Equation (1.2)]. The grid cell sums the input from several HD band cell populations selective for different heading directions and the result is spatially periodic firing at equidistant points across the environment. An analogy can be drawn between this mechanism of representing space and hypotheses regarding hierarchical representation of visual input by visual cortices (Grill-Spector and Malach, 2004). Neurons in early visual areas are selective for basic elements that make up a visual scene: orientation, spatial frequency, color etc. The output of these neurons is then combined into successively higher level, conjunctive representations by downstream neurons. Similarly, in the entorhinal cortex an animal's position in 3D space might be represented as the combination of firing activity from neurons selective for perceived modulo displacement in a particular direction.

There is evidence for neurons in the brain that present several of the characteristic features required of the HD band cell populations in our model. Neurons that are selective for an animal's head direction have been discovered in various regions of the brain, including postsubiculum and thalamic areas (Taube et al., 1990a,b; Taube, 2007). These cells use environmental cues to determine head direction and so constitute an allocentric frame of reference for the animal. They have been shown to be selective for heading directions within ±45° of their preferred direction on average (Taube, 2007), however cells with broader directional selectivity, around ±90° have also been reported (Sargolini et al., 2006; Brandon et al., 2011, 2013) and this is the range of selectivity of the HD band cell populations in our model [refer to Equation (1.5)]. There are reports of HD selective cells that are also theta rhythmic in their firing (Cho and Sharp, 2001; Cacucci et al., 2004; Brandon et al., 2011, 2013; Tsanov et al., 2011). The firing rate of HD cells in entorhinal cortex has been shown to be modulated by running speed (Sargolini et al., 2006), as is required by the oscillatory interference mechanism of path integration if grid fields are to maintain their spacing as an animal's speed varies. Cells which fired only when a rat ran in particular direction and whose firing was modulated by the animal's running speed have also been reported in the medial septum (King et al., 1998). Welday et al. (2011) observed neurons in hippocampus, medial septum and anterior thalamus which fired in theta frequency bursts that varied as the cosine of a rat's allocentric direction, also a feature of the oscillatory interference mechanism. There is also evidence of cells whose firing rate maps resemble bands across an environment, similar to those produced by the HD band cell populations in our model (Krupic et al., 2012). Krupic et al. (2012) found cells in MEC and para-subiculum that produced such firing rate maps and seemed to be able to switch from participation in ensembles generating band patterns to ensembles generating grid patterns. Finally, there is evidence of cells in the medial septum and diagonal band of Broca that are phase locked to different preferential phases of a theta frequency rhythm, which could provide the temporal baseline phase offsets in our model (King et al., 1998). It is also interesting to note that cells which can switch between grid cell firing and band cell firing patterns have been observed (Krupic et al., 2012); similarly in our model [and Burgess (2008)], if the grid cell were to only receive input from one of the HD band cell populations it too would revert from a pattern of grid firing to a pattern of band firing (results not shown).

The value of the leak time constant in our model was chosen to best fit the available experimental data. A value of 0.1 s allows the model to generate DC shifts with amplitude values in a similar range to those observed *in vivo*. It also produces values of the difference in DC shift amplitude and membrane potential theta oscillation (MPO_θ_) amplitude, in-field vs. out-of-field, which match the *in vivo* data well (this is presented in detail in the Results section above). There are several justifications for using such a lengthy decay time constant. Whilst the apparent membrane time constant for stellate cells in MEC layer II has been established from sharp electrode recordings and whole cell patch recordings to be 0.005–0.015 s (Garden et al., 2008; Pastoll et al., 2012), for pyramidal cells, which also demonstrate grid field tuning, the apparent value of the leak time constant is longer: Fernandez and White (2009) report values of 0.066 ± 0.006 s for pyramidal cells in MEC layer III exhibiting spike frequency adaptation and 0.034 ± 0.005 s for non-adapting pyramidal cells. We have endeavored to keep the mathematical description of our model simple, using a basic leaky integrate-and-fire model framework so that it is clear how DC shifts in-field arise. However, a more biophysically realistic model could clarify the exact mechanisms that lead to an increased membrane time constant and corresponding increase in the integration period of the grid cell. Potential mechanisms include the activity of NMDA receptors, which are known to have a long decay time constant (up to 0.2 s) (Edmonds et al., 1995), or the addition of a calcium-activated non-specific cation (CAN) current, leading to afterdepolarizations (Egorov et al., 2002; Magistretti et al., 2004). Both of these mechanisms have been explored previously in the context of persistent firing during delay periods in working memory tasks (Wang, 1999; Fransen et al., 2002, 2006; Miller et al., 2003), however a more detailed biophysical model of this type is beyond the scope of this paper. Finally, there is evidence from *in vitro* studies made in all layers of MEC that stimulation via afferents from pre- and para-subiculum can result in long decay times for intracellularly recorded synaptic potentials. For example, stimulation received from pre-subiculum produced the following decay times in entorhinal cortex: 0.068 ± 0.008 s in layer III, 0.069 ± 0.007 s in layer V and 0.092 ± 0.009 s in layer VI (Canto et al., 2012). In the same study stimulation from para-subiculum produced the following decay times: 0.085 ± 0.014 s in layer III and 0.075 ± 0.011 s in layer VI. If stimulation was delivered at a frequency of 10 Hz then these decay times were altered: layer III exhibited decay times of 0.095 ± 0.014 s following pre-subicular stimulation, whilst layer VI exhibited a decay time of 0.157 ± 0.044 s. Following para-subicular stimulation at 10 Hz layer III showed a decay time of 0.088 ± 0.016 s, layer V showed a decay time of 0.093 ± 0.033 s and layer VI showed a decay time of 0.103 ± 0.015 s. Since cells in pre- and para-subiculum demonstrate head direction tuning they are well placed to correspond with the HD cell population inputs in our model. MEC layer II cells did not demonstrate such lengthy decay times in the same study, however it could be possible that these cells still display grid field tuning due to inputs from other MEC neurons but that they function to transmit this information to downstream structures, whilst the path integration calculations underlying grid field tuning are performed in other layers that do demonstrate the necessary longer decay times and integration periods.

The fit between the dynamics of our model and the empirical data depends upon the value of several parameters. Of particular importance are the time constant of the leak conductance in the grid cell membrane [τ_GL_, see Equation (1.1)] and the baseline temporal phase offsets which exist between the HD populations [φ_offset_n_, refer to Equation (1.2)]. When τ_GL_ is very short, integration is not sufficient given the theta frequency incoming HD population inputs to generate large amplitude DC-shifts within field. The presence of “gaps” of ½ a theta cycle period (due to half-wave rectification) when the grid cell receives no input means that any integrated potential in the membrane is lost to the leak conductance and there is no increase in depolarization or subsequent increase in DC-shift. This can be counteracted to some degree by the use of optimally spread baseline temporal phase offsets of the input, as we have used here. When τ_GL_ is longer (~100 ms) however the effect of widely spread φ_offset_n_, values is less important in order to see DC shifts, since integrated input persists for the duration of the “gaps” in the HD population input. With the value of τ_GL_ we have used here it is possible to set all φ_offset_n_ values to zero and still see large DC shifts within field (results not shown). Interestingly, when all φ_offset_n_ values are zero, since the HD population inputs to the grid cell are more synchronized (particularly within field) the grid cell produces larger amplitude MPO_θ_ oscillations that bear some resemblance to the “large theta” cells recorded by Domnisoru et al. (2013).

It was not our goal to find and report the best possible fit to the empirical data in this manuscript. This work was intended to serve as a proof of concept that DC-shifts can be generated by an oscillatory interference model of grid cell generation and to identify the key concepts for generating this behavior. Searching for a parameterization that achieves the best fit to empirical data remains a possibility for future research. However, the use of a τ_GL_ value = 100 ms does produce a reasonably good fit between our model and the empirical results reported in Domnisoru et al. (2013), Schmidt-Hieber and Häusser (2013) (specifically the results we present in Figures 2D, 9) and so this suggests itself as a prediction of our model: that grid cells with long decay constant values are required to perform the path integration calculations underlying grid cell firing. Cells in entorhinal cortex layer II which have been shown to possess much shorter effective decay constants (Garden et al., 2008; Canto et al., 2012; Pastoll et al., 2012) might potentially receive input from path integration calculating grid cells and then act as transmitters of this information but not actually perform path integration of HD band cell inputs themselves. Alternatively, layer II cells might still perform path integration with much shorter decay constants but receive additional input during within field periods from grid cells in other layers that do demonstrate the necessary longer decay constants; hence producing large amplitude DC shifts within field.

Although the influence of noise in the VCO inputs was not explicitly analyzed in the context of this study, it is worth noting that the magnitude of τ_GL_ could influence the effect of VCO input noise on the spatial tuning of the simulated grid cell. We speculate that such an analysis would show that τ_GL_ may gate a speed / accuracy trade-off in the spatial precision of the grid cell's firing (Wickelgren, 1977). That is, larger values of τ_GL_ may reduce the influence of noise on the grid cell as rapid fluctuations would have less overall impact given longer windows of integration. However, large increases in the temporal integration window would also reduce the temporal precision with which the grid cell could feasibly track the movement of the animal (represented by the VCO inputs) and thus would have the influence of blurring the boundaries of the firing fields.

It is worth noting that the ability to increase τ_GL_ until the window of integration is on a similar time scale to the duration of a theta cycle is only possible in our model because of our grid cell's reduced dependence on performing “coincidence detection” in the VCO inputs, in contrast to previously described oscillatory interference models. In earlier variants (Burgess et al., 2007; Hasselmo, 2008) each VCO provided input to the grid cell on every theta cycle (modulo some phase shift) and the function of the grid cell was to perform coincidence detection so as to only fire when all of the inputs were in phase. If a long window of integration were added to the grid cell in such a model, the spatial firing pattern would be degraded, since sub-theta cycle precision is required to distinguish the informative phase shifts in each cycle. In our model the oscillatory interference mechanism occurs in the HD band cells as opposed to the grid cell (this possible variation of the oscillatory interference model was discussed in Burgess, 2008), so the time constant of our grid cell is not constrained to be less than a theta cycle. In our model the HD band cells do not provide input to the grid cell on every theta cycle, they only contribute input when the animal is moving in a direction within 90° of that population's preferred heading direction and the amplitude of that input determines the position in the environment relative to the characteristic bands the HD band cells produce (refer to Figure 2C). The grid cell in our model is required to detect the overall level of input from the HD band cell populations; the summation of these inputs is larger when phase differences between them are small but detection of coincidence between individual cycles in not required since the information is contained in the amplitude of the HD band cell populations' activity.

Our results replicate the same trends evident in the experimental data when subjected to the same analyses used in Domnisoru et al. (2013), Schmidt-Hieber and Häusser (2013) (refer to Figure 9). However, within our data the correlation between MPO_θ_ oscillation amplitude and firing rate is more similar in magnitude to that observed between DC-shift amplitude and firing rate and is statistically significant. Also, the difference between the amount of information regarding in-field position demonstrated by the DC-shift time series and the MPO_θ_ oscillation amplitude time series is reduced in our data relative to that observed in the empirical data. The differences in the correlation values are influenced by the fact that we calculated the correlations using unbinned data. It is also possible that the high amplitude local field theta rhythms observed in the extracellular potentials in intact animals (such as those studied by Domnisoru and Schmidt-Hieber) could increase the apparent mean out-of-field MPO_θ_ amplitude through ephaptic coupling (Anastassiou et al., 2010, 2011; Fröhlich and McCormick, 2010). Such coupling would reduce the difference in MPO_θ_ amplitude between in-field vs. out-of-field epochs, thereby reducing the correlation and MI between MPO_θ_ amplitude and firing rate. We have not included such coupling in our model, however we tested simulations in which we added an additional sinusoid of a slightly different frequency (frequency of 6.5 Hz for added sinusoid vs. baseline frequency of 6 Hz) to the membrane potential of our grid cell to simulate a local field rhythm and found correlation values which more closely resemble those observed in the empirical data. For these experiments, binned correlation values produced statistically significant DC-shift *r* values around 0.9 and MPO_θ_ oscillation *r* values around 0.5 but which fail to reach significance, similar to the results reported by Schmidt-Hieber and Häusser (2013) (data not shown).

The phase precession effect demonstrated by grid cells (and place cells) in the hippocampal formation presents a convincing example of a phase coding scheme being implemented in the brain to represent temporal sequences of events. The relative timing of several grid cells' action potentials relative to one cycle of a shared baseline theta rhythm can be indicative of which grid field was just visited, which is closest to the animal's current position and which is being approached on the current trajectory (O'Keefe and Recce, 1993; Jensen and Lisman, 1996). Phase precession is an integral feature of oscillatory interference models of grid cell formation but not continuous attractor network models. We have illustrated here that this remains a feature of our simulations (see Figure 8), as well as having been observed in *in vivo* recordings (Domnisoru et al., 2013; Schmidt-Hieber and Häusser, 2013).

Our model predicts that lengthening of the time constant of the leak conductance (τ_GL_) should produce larger amplitude DC-shifts in-field; if τ_GL_ is too long and disproportionate to the firing threshold level of the grid cell then grid fields will begin to merge, as the leak conductance is not fast enough to lower the membrane potential to its baseline value when the animal is between fields. In this case the grid cell would become a persistent spiking cell (see Figure 7B). It is likely that other membrane channel dynamics that have not been included in our simple model could act to counter-balance such an increase in τ_GL_ and bring the membrane potential back to baseline but the entire dynamic of the system would be very different and therefore the pattern of grid fields would certainly be disrupted. Our model also predicts that the value of the temporal phase offsets between the baseline oscillations for the HD band cell populations (φ_offset_n_) can influence the amplitude of the theta oscillations produced by the grid cell. If φ_offset_n_ are all close to zero then the cell will behave like a “large theta cell,” producing more consistent, large amplitude MPO_θ_ 's that ride on top of a DC voltage trace that varies with position relative to the grid fields. If τ_GL_ is short then widely spaced φ_offset_n_ can allow the grid cell to still perform successful integration and produce changes in DC value within field, although these will be lower in amplitude. Reliably maintained temporal phase offsets [such as those shown in King et al. (1998)] could be achieved using ring attractor networks of cells acting as oscillators (Blair et al., 2008).

In summary, this work has shown that it is possible for an oscillatory interference mechanism of path integration to also generate increases in the DC membrane potential level of grid cells within field. The model is similar to the (Burgess, 2008) model of grid cell formation, based on directional VCO inputs from separate neurons impinging on a grid cell, but with the following modifications: (i) the leaky integrate-and-fire dynamics incorporate a long decay time constant, (ii) the baseline oscillations for each of these upstream populations has the same frequency but some temporal phase offset, to allow for temporal spread in the inputs to the grid cell, which can then be integrated, and (iii) the inputs to the grid cell are assumed to be from upstream populations of excitatory cells and as such are half-wave rectified, to represent a change in average firing rate activity and to provide a purely excitatory input to the grid cell with a non-linear mean value. Our model demonstrates that the oscillatory interference mechanism of path integration cannot be ruled out as possibly underlying grid cell formation based on the current evidence.

### Conflict of interest statement

The authors declare that the research was conducted in the absence of any commercial or financial relationships that could be construed as a potential conflict of interest.
